# Variation in the bovine FABP4 gene affects milk yield and milk protein content in dairy cows

**DOI:** 10.1038/srep10023

**Published:** 2015-06-12

**Authors:** H. Zhou, L. Cheng, W. Azimu, S. Hodge, G. R. Edwards, J. G. H. Hickford

**Affiliations:** 1 Department of Agricultural Science, Faculty of Agricultural and Life Sciences, Lincoln University, Lincoln 7647, New Zealand; 2 College of Animal Science, Tarim University, Alar, Xinjiang Province 843300, China

## Abstract

Fatty acid binding proteins (FABPs) bind long-chain fatty acids and are involved in their intracellular transport. Of the known bovine FABP genes, *FABP4* has been mapped to a region on chromosome 14 that contains quantitative trait loci for milk traits. This study investigated the association of *FABP4* haplotypes with milk production traits in 719 Holstein-Friesian × Jersey cows. Polymerase chain reaction-single strand conformational polymorphism (PCR-SSCP) analysis of a variable region of the gene revealed three haplotypes (*A*, *B* and *C*). Five single nucleotide polymorphisms (SNPs) were identified: two in exon 3 and three in intron 3. *A* was associated (*P* = 0.032) with increased milk protein percentage (present: 4.00 ± 0.02%; absent: 3.95 ± 0.02%) and *B* was associated (*P* = 0.009) with increased milk yield (present: 23.81 ± 0.23 kg/d; absent: 23.06 ± 0.21 kg/d), but tended to be associated with a decrease in protein percentage and an increase in protein yield. Cows with genotypes *AA*, *AB* and *AC* produced less milk, but with a higher protein percentage than *BC* cows. This suggest that *FABP4* affects milk yield and milk protein content, both economically important traits, and that further study of this gene is warranted.

Fatty acid binding proteins (FABPs) bind long-chain fatty acids and transport them within cells. They have also been implicated in lipid metabolism, the expression of fatty acid-responsive genes and the maintenance of cell membrane fatty acid levels^1^,^2^.

There are nine known members of the *FABP* gene family (*FABP1*-*FABP9*) and they are differentially expressed in different tissues^3^. In the bovine mammary gland, the most abundant FABP mRNAs are for FABP3, FABP4, and FABP5; and for all, gene expression is greatly up-regulated during lactation^4^.

*FABP4* has been mapped to BTA14^5^ in a location (46,833,665-46,838,053) that is rich in quantitative trait loci (QTL) for milk production traits^6^,^7^. This suggests it may be a candidate gene or marker for milk production. Various effects of *FABP4* on milk production and quality have recently been described^8^,^9^. Marchitelli *et al.*^8^ found that *FABP4* influenced both medium chain and long chain fatty acid (FA) levels in milk, and suggested *FABP4* was the “most important” gene affecting milk fat composition out of nine candidate genes that they studied. Similarly, Nafikov *et al.*^9^ reported that certain *FABP4* haplotypes were associated with particular fatty acid profiles in milk, but found no differences in milk yield.

*FABP4* is comprised of four exons separated by three introns. Variation in bovine *FABP4* has been investigated in both beef cattle^10^,^11^,^12^ and dairy cattle^8^,^9^. Eight single nucleotide polymorphisms (SNPs) have been validated in beef cattle, and of these, five SNPs (one non-synonymous SNP, one splice-site SNP and three intronic SNPs) are located in a small exon 3-intron 3 region^11^ that would be suitable for polymerase chain reaction-single strand conformational polymorphism (PCR-SSCP) analysis analysis.

The purpose of this investigation was to take advantage of the PCR-SSCP technique to genotype the variable region of *FABP4* in a large herd of 719 Holstein-Friesian × Jersey cross dairy cows grazed in an outdoor grass-fed New Zealand dairy system, and to investigate whether there is any association between variation in *FABP4* and major milk production traits.

## Results

### Variation in bovine *FABP4* and haplotype frequencies

**Figure 1:**
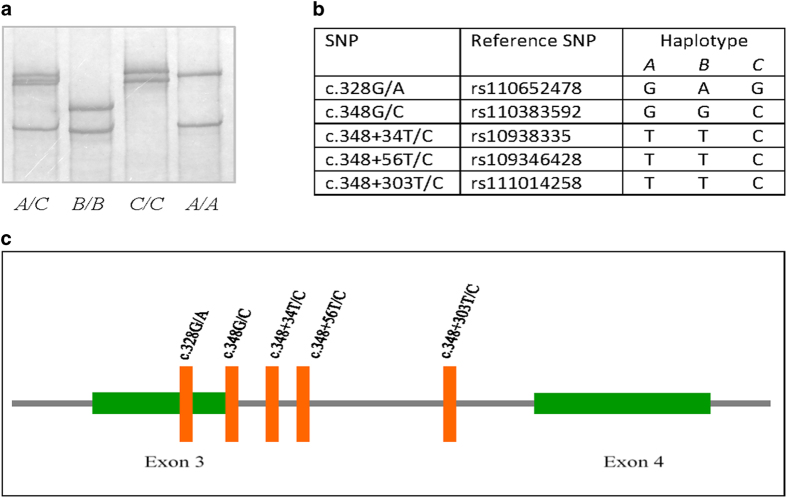
Variation in the bovine FABP4 gene (*FABP4*). (**a**) Three PCR-SSCP banding patterns representing (**b**) three haplotype sequences (*A, B* and C) were detected in the exon 3–intron 3 region. (**c**) Of the five SNPs identified, two were located in exon 3 and three were in intron 3. The nucleotide numbering follows the HGVS nomenclature ( www.hgvs.org/mutnomen).

PCR-SSCP analysis of the H-F × J cross dairy cows revealed three different PCR-SSCP banding patterns for *FABP4* (Fig. 1a). Sequencing of PCR amplicons representative of these different SSCP patterns revealed three different DNA sequences. Five SNPs were identified among these sequences, with two found in exon 3 and three found in intron 3 of the gene. Of the two exon SNPs, one was non-synonymous (c.328 G/A) and would notionally result in an amino acid substitution (p.V110M), while the other was a synonymous SNP (c.348G/C) at the last nucleotide position of exon 3. This splice-site SNP was in complete linkage with the three intron SNPs based on the contiguous sequences that were obtained (Fig. 1b). Together, these five SNPs define three haplotype sequences (named haplotypes *A*, *B* and *C*).

In the dairy cows investigated, six genotypes were found, at frequencies of 22.1% (*AA*), 16.1% (*AB*), 32.8% (*AC*), 3.2% (*BB*), 12.0% (*BC*) and 13.8% (*CC*). The haplotype frequencies were 46.6%, 17.2% and 36.2% for *A*, *B* and *C* respectively. The *P*-value for the chi-square for deviation from HWE was 0.968, suggesting the ‘population’ is at equilibrium.

### *FABP4* variation and milk production traits

Individual cow milk yields ranged from 12.2 kg/d to 33.5 kg/d, with an average of 22.6 kg/d. The protein percentages ranged from 3.31% to 4.74%, with an average of 4.03%. The fat percentages ranged from 3.52% to 6.64%, with an average of 5.01%.

The presence of *A* was associated (*P* = 0.032) with an increase in protein percentage (present: 4.00 ± 0.02%; absent: 3.95 ± 0.02%), while the presence of *B* was associated (*P* = 0.09) with an increase in milk yield (present: 23.81 ± 0.23 kg/d; absent: 23.06 ± 0.21 kg/d), and tended to be associated with decreased protein percentage but increased protein yield (Table 1). The increase in protein yield associated with *B* appeared to be primarily due to the increase in the amount of milk produced. A trend of association with increase milk yield, but decreased protein percentage was also observed for the presence of *C* (Table 1).

There were significant differences among the five genotypes tested (*AA, AB, AC, BC* and *CC*) for milk yield (F_4, 670_ = 5.44; *P* < 0.001) and protein percentage (F_4, 670_ = 5.85; *P* < 0.001) (Table 2). From pairwise comparisons using Tukey’s HSD (*P* < 0.05), cows with genotype *BC* produced significantly more milk than any other genotype tested. For milk protein percentage, all three genotypes containing the *A* haplotype (*AA, AB* and *AC*) were significantly higher than *BC* cows, but *CC* cows were not different to either *AA*, *AB*, *AC* or *BC* cows.

## Discussion

All the SNPs that have been previously reported in the amplified region in beef cattle^9^,^12^ or dairy cattle^8^ were detected in the H-F × J cross dairy cows in this study. However, the linkage between the SNPs observed in the dairy cattle investigated here appears to be different to that seen in beef cattle^10^. In beef cattle the splice-site SNP is in linkage disequilibrium with two of the intron SNPs, whereas in the HF × J dairy cows described in this study, the splice-site SNP appears to be in complete linkage with all three of the intron SNPs. The linkage of the splice-site SNP and two intron SNPs was also observed by Nafikov *et al.*^8^ in their haplotype 1 (H1) cows, although they do not describe what breed, or breeds of dairy cow were being investigated. Together this would suggest that greater genetic variation may exist in the whole gene than is suggested by SNP analysis of one small region and/or that more cattle of a diversity of breeds need to be studied to better resolve the genetic variation in *FABP4*.

This splice-site SNP, although synonymous, may have a functional effect as it has been reported that variation at the last nucleotide position of exons may have an effect on the function of donor splice-sites and may result in exon-skipping, partial exon deletion, or intron retention due to activation of cryptic splice-sites^13^. The potential for this will need to be tested in future functional studies, and in both beef and dairy cattle.

On the basis of SNP comparison, the *FABP4* haplotypes *A*, *B* and *C* described here appear to match H2, H3 and H1 respectively as described by Nafikov *et al.*^9^. Nafikov *et al.*^9^ reported that *FABP4* haplotype H3 was associated with lower saturated fatty acid (SFA) concentrations, a lower SFA: unsaturated fatty acid (UFA) ratio and lower levels of lauric (12 carbon) and myristic (14 carbon) acid; but higher levels of UFA and monounsaturated fatty acid (MUFA) when compared with H1 during the first three months of lactation. No effect was recorded on overall milk production or milk fat levels. They suggested that the FABP4 protein controlled compositional change in milk, which they attributed to the lipolysis of adipose tissue as opposed to *de novo* FA synthesis.

While not directly equivalent, the results obtained by Nafikov *et al.*^9^ may compare with those in this study, where the presence of haplotype *B* (H3) appears to be associated with increased milk yield, increased protein yield and increased milk solids yield. The presence of *B* does not appear to affect milk composition with regard to the fat percentage, but there was some indication that it may cause a small, but statistically significant, reduction in protein percentage. This difference may however reflect differences in the production system as the NZ HF × J cows were farmed outdoors on pasture and typically produce less milk than US cows farmed indoors on different nutritional regimes.

Marchitelli *et al.*^8^ defined two alleles of *FABP4* based on the analysis of four SNPs. Two of their SNPs match those defined in this study, with g.3711G>C equivalent to c.348G/C and g.3745T>C equivalent to c.348 + 34T/C. This would suggest that their “minor allele” corresponds to haplotype *C* in this study and their major allele corresponds to both haplotypes *A* and *B*. If this assessment is correct then direct comparison of the results of the two studies is somewhat compromised. However, our results indicate that the presence of the *B* haplotype when comparing cows of *CC* genotype with cows of *BC* genotype would appear to be associated with an increase in milk yield, protein yield and milk solid yield (this also being consistent with the absence/presence models - Table 1). The presence of the haplotype *A* (when comparing cows of *AA*, *AB* and *AC* genotypes with cows of *BC* genotype), would appear to be associated with decreased milk yield, but increased protein percentage. Thus, assuming a comparison can be drawn between our results and those of Marchitelli *et al.*^8^, haplotypes *A, B* (equivalent to the “major allele”) and *C* (equivalent to the “minor allele”) all appear to be affecting milk traits. This comparison is however weakened by the fact that there are no difference between cows with the *CC* genotype and cows with geno-types containing haplotype *A*. It must also be acknowledged that Marchitelli *et al.*^8^ were studying small numbers of cows of different breed and that they did not analyse any milk traits that can be compared easily with those measured in this study. An appropriate conclusion would seem to be that at best both studies reveal *FABP4* variation is affecting milk traits; and overall, as suggested earlier in this discussion, more needs to be known about the extent of genetic variation in different breeds, and cows of the same breed, before it can be claimed that the effects seen are real.

**Figure 2:**
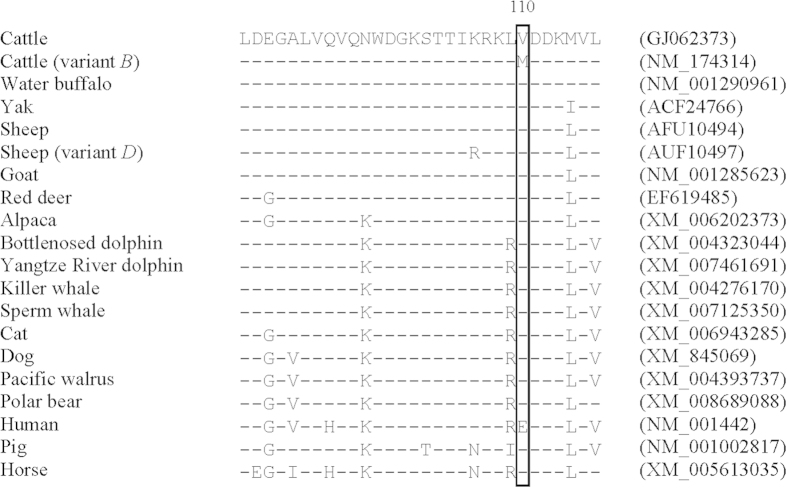
Alignment of amino acid sequences for *FABP4* exon 3 reveals that residue 110 is conserved across species and that the substitution V110M appears to be unique to cattle.

It is notable that the *B* haplotype associated with increased milk protein percentage carries the nucleotide substitution c.328G> A that would lead to the putative amino acid substitution of valine to methionine at residue 110, the last residue of β-strand H. This residue is relatively conserved across species, and the substitution to methionine is rare and it appears to be unique to cattle (Fig. 2). The FABP4 protein comprises ten antiparallel β-barrels inside which a solvent-accessible ligand-binding pocket is located^14^. The potential effect of this substitution on protein structure and function requires further investigation.

It is interesting to note that the New Zealand dairy cows investigated in this study had a higher frequency (47% versus 27%) of haplotype *A* (H2) that favours protein percentage, but a lower frequency (17% versus 24%) of haplotype *B* (H3) that favours milk yield, compared to the cows studied in Nafikov *et al.*^9^. The cows studied in Nafikov *et al.*^9^ were derived from the Iowa State University Dairy Herd ( http://www.ans.iastate.edu/centers/dairy/) and the Kansas State University Dairy Herd ( http://www.asi.k-state.edu/facilities/Dairy-Unit.html). The Iowa State University Dairy cows have an average milk yield of 36 kg/d and average protein percentage of 3% ( http://www.ans.iastate.edu/centers/dairy/). While this is not fully consistent with the lower average milk yield (22.6 kg/d) and a higher average protein percentage (4%) for the New Zealand cows investigated in this study, it needs to be remembered that the New Zealand cows were exclusively pasture fed and that the New Zealand Dairy Industry’s Breeding Worth (BW) genetic evaluation system ( http://www.dairynz.co.nz/animal/animal-evaluation/about-nzael/) favours genetics that produces a higher protein percentage, but lower volume of milk.

We are also aware that sire identity has not been factored into the GLMs and thus that sire may be a confounding factor in this study. While sire could be ascertained for some cows, the majority of them were of unknown paternity as sire genetics (semen straws) were purchased from a commercial semen producer on the basis of quantitative genetic evaluation of performance for key dairy traits. These straws often contain mixed semen, so individual sire identity would be impossible to ascertain. However, haplotype frequencies were in HWE across all cow ages, suggesting no individual sire confounded the results. Additionally, this weakness in the data can be partially mediated in two further ways. Firstly, the cows studied ranged in age between 3 and 9 years and, due to the improbability of the same sires being used in the production of semen straws over this period, are unlikely to share paternity. Secondly, if we concede that cows of the same age may share paternity, the inclusion of cow age in the GLMs as an explanatory variable, would have at least partially accommodated this effect.

The possibility that the effects seen in this study may be due to the presence of other genes cannot be ruled out, especially if there are other genes that affect milk traits in proximity to *FABP4* on BTA14. However, taken together these findings support the contention that *FABP4* affects economically important milk traits and suggest the function of this gene in dairy cattle is worthy of further investigation.

## Materials and methods

All research involving animals were carried out in accordence with the Animal Welfare Act 1999 (New Zealand Govertment) and approved by the Lincoln University Animal Ethics Committee (AEC Number 521).

### Animals and DNA samples

Genetic variation in bovine *FABP4* was investigated in 719 Holstein-Friesian × Jersey (HF × J) dairy cows that had commenced lactation within ten weeks of the study. The cows were between three to nine years old (i.e 2^nd^ to 8^th^ parity). All cows were maintained on the Lincoln University Dairy Farm (LUDF; Canterbury, New Zealand) and grazed over their lactation on mixed ryegrass/white clover pasture. At commencement of the study a blood sample from each cow was collected onto an FTA card and allowed to air dry. Genomic DNA was purified from a 1.2 mm punch of the dried blood spot using a two-step washing procedure as described by Zhou *et al.*^15^.

### Milk sampling and phenotype measurement

All cows were milked twice daily and the daily milk yield in kilograms wet weight was recorded using Tru-test milk meters (Tru-test Ltd; New Zealand). Milk samples for analysis were collected once a month for a period of 6 months starting from September to February. These samples were analysed for fat percentage (%) and protein percentage (%) using Fourier-Transform Infra-Red Spectroscopy (MilkoScan FT 120 Foss, Hillerød, Denmark). These concentrations and the milk yield for that day were used to calculate fat yield (kg/d), protein yield (kg/d) and milk solid yield (kg/d).

### PCR-SSCP analysis and genotyping of *FABP4*

Two PCR primers, ACTTAGATGAAGGTGCTCTG and CCTCAGGACTAAACAACTTATG, were designed based on a bovine *FABP4* sequence ENSBTAG00000037526, to amplify a 525 bp fragment spanning exon 3 and part of intron 3 of the gene. The primers were synthesised by Integrated DNA Technologies (Coralville, IA, USA).

PCR amplification was performed in a 15 μL reaction containing the genomic DNA on one 1.2 mm punch of FTA paper, 0.25 μM of each primer, 150 μM of each dNTP (Bioline, London, UK), 2.5 mM of Mg^2+^, 0.5 U of Taq DNA polymerase (Qiagen, Hilden, Germany) and 1 × reaction buffer supplied. The thermal profile consisted of 2 min at 94 ^o^C, followed by 35 cycles of 30 s at 94 ^o^C, 30 s at 60 ^o^C and 30 s at 72 ^o^C, with a final extension of 5 min at 72 ^o^C. Amplification was carried out in S1000 thermal cyclers (Bio-Rad, Hercules, CA, USA).

Amplicons were visualized by electrophoresis in 1% agarose gels (Quantum Scientific, Queensland, Australia), using 1 × TBE buffer (89 mM Tris, 89 mM boric acid, 2 mM Na_2_EDTA) containing 200 ng/mL of ethidium bromide.

A 0.7 μL aliquot of each amplicon was mixed with 7 μL of loading dye (98% formamide, 10 mM EDTA, 0.025% bromophenol blue, 0.025% xylene-cyanol). After denaturation at 95 ^o^C for 5 min, the samples were rapidly cooled on wet ice and then loaded on 16 cm × 18 cm, 14% acrylamide:bisacrylamide (37.5:1) (Bio-Rad) gels. Electrophoresis was performed using Protean II xi cells (Bio-Rad), at 350 V for 18 h at 4 ^o^C in 0.5 × TBE buffer. Gels were silver-stained according to the method of Byun *et al.*^16^.

### Sequencing of haplotypes and sequence analysis

PCR amplicons representing different banding patterns from cows that appeared to be homozygous were sequenced in both directions at Lincoln University, New Zealand. Sequence alignments, translations and comparisons were carried out using DNAMAN (version 5.2.10, Lynnon BioSoft, Vaudreuil, Canada).

### Statistical analyses

Hardy-Weinberg equilibrium (HWE) for the *FABP4* genotypes was analysed using an online chi-square test ( http://www.had2know.com/academics/hardy-weinberg-equilibrium-calculator-3-alleles.html).

In general, the statistical treatment of the data followed that described in Hickford *et al.*^17^. All statistical analyses were performed using Minitab version 16 (Minitab Inc., PA, USA). General Linear Models (GLMs) were used to assess the presence or absence of each of three *FABP4* haplotypes separately on six milk traits: milk yield, fat percentage, protein percentage, fat yield, protein yield and milk solid yield. For milk traits where more than one haplotype had an effect with statistical significance of *P* < 0.2 in the “single-haplotype” GLMs (and could thus potentially impact on the milk trait being tested), a second series of “multi-haplotype” GLMs that included these haplotypes, was then performed.

For those milk traits that were found to be significantly associated with the presence or absence of any haplotypes, comparisons between genotypes were then performed by GLM, with pairwise comparisons of genotypes carried out using Tukey’s Honestly Significant Difference (HSD) test to account for the multiple comparisons being made. Only genotypes with a frequency of greater than 5% (thus represented by an adequate sample size), were included in these analyses.

Cow age and ‘weeks in milk’ (counted from the day of calving) were correlated with many of the measured milk traits and so were included in all of the GLMs as explanatory factors (Table 3).

## Additional Information

**How to cite this article**: Zhou, H. *et al.* Variation in the bovine FABP4 gene affects milk yield and milk protein content in dairy cows. *Sci. Rep.* **5**, 10023; doi: 10.1038/srep10023 (2015).

## Figures and Tables

**Table 1:** Association of presence or absence of *FABP4* haplotypes with various milk production traits (mean ± SE)^1,2^.

**Trait**^3^	**Haplotype assessed**	**N**		**Single-haplotype model**			**Multi-haplotype model**			
		**Absent**	**Present**	**Absent**	**Present**	***P***	**Other haplotypes in model**	**Absent**	**Present**	***P***
MY (kg/d)	*A*	208	511	23.77 ± 0.24	23.07 ± 0.17	**0.005**	*B,C*	23.56 ± 0.26	23.30 ± 0.19	0.390
	*B*	494	225	23.07 ± 0.17	23.73 ± 0.24		*A,C*	23.06 ± 0.21	23.81 ± 0.23	**0.009**
	*C*	298	421	22.99 ± 0.20	23.44 ± 0.18	*0.063*	*A,B*	23.17 ± 0.24	23.70 ± 0.20	*0.058*
FP (%)	*A*	208	511	4.97 ± 0.04	5.06 ± 0.03	**0.032**	*B,C*	4.99 ± 0.05	5.04 ± 0.03	0.397
	*B*	494	225	5.06 ± 0.03	4.98 ± 0.04	*0.090*	*A,C*	5.05 ± 0.04	4.98 ± 0.04	0.115
	*C*	298	421	5.07 ± 0.04	5.01 ± 0.03	0.154	*A,B*	5.04 ± 0.04	4.98 ± 0.03	0.208
PP (%)	*A*	208	511	3.93 ± 0.02	4.01 ± 0.01	****<0.001****	*B,C*	3.95 ± 0.02	4.00 ± 0.02	
	*B*	494	225	4.00 ± 0.01	3.96 ± 0.02		*A,C*	3.99 ± 0.02	3.95 ± 0.02	*0.074*
	*C*	298	421	4.02 ± 0.02	3.97 ± 0.02	**0.009**	*A,B*	3.99 ± 0.02	3.95 ± 0.02	*0.073*
FY (kg/d)	*A*	208	511	1.17 ± 0.01	1.16 ± 0.01	0.162	*B*	1.17 ± 0.01	1.17 ± 0.01	0.293
	*B*	494	225	1.16 ± 0.01	1.17 ± 0.01	0.167	*A*	1.16 ± 0.01	1.18 ± 0.01	0.304
	*C*	298	421	1.15 ± 0.01	1.17 ± 0.01	0.200				
PY (kg/d)	*A*	208	511	0.93 ± 0.01	0.92 ± 0.01	0.257				
	*B*	494	225	0.92 ± 0.01	0.93 ± 0.01	0.055				
	*C*	298	421	0.92 ± 0.01	0.93 ± 0.01	0.401				
MSY (kg/d)	*A*	208	511	2.10 ± 0.02	2.08 ± 0.01	0.172	*B*	2.10 ± 0.02	2.09 ± 0.01	0.359
	*B*	494	225	2.08 ± 0.01	2.10 ± 0.02	0.085	*A*	2.08 ± 0.02	2.11 ± 0.02	0.163
	*C*	298	421	2.07 ± 0.01	2.09 ± 0.01	0.247				

^1^Predicted means and standard error from GLM including ‘cow age’ and ‘weeks in milk’ as factors.

^2^*P* < 0.05 in bold and 0.05 ≤ *P* < 0.1 in italics.

^3^MY–milk yield; FP–fat percentage; PP–protein percentage; FY–fat yield; PY–protein yield; MSY–milk solid yield.

**Table 2:** The effect of *FABP4* genotype on various milk production traits (Mean ± SE)^1,2^.

**Genotype**							
**Trait**^3^		***AA***	***AB***	***AC***	***BC***	***CC***	***P***
	N	**159**	**116**	**236**	**86**	**99**	
MY (kg/d)		22.93 ± 0.26^b^	23.10 ± 0.30^b^	23.56 ± 0.22^b^	24.71 ± 0.35^a^	23.08 ± 0.32^b^	<0.001
FP (%)		5.07 ± 0.05^a^	5.06 ± 0.05^a^	5.05 ± 0.04^a^	4.85 ± 0.06^b^	5.04 ± 0.06^ab^	0.024
PP (%)		4.03 ± 0.02^a^	4.00 ± 0.03^a^	4.01 ± 0.02^a^	3.88 ± 0.03^b^	3.95 ± 0.03^ab^	<0.001
FY (kg/d)		1.15 ± 0.01	1.16 ± 0.01	1.16 ± 0.01	1.20 ± 0.02	1.16 ± 0.01	0.157
PY (kg/d)		0.92 ± 0.01^ab^	0.92 ± 0.01^ab^	0.93 ± 0.01^ab^	0.96 ± 0.01^a^	0.90 ± 0.01^b^	0.011
MSY (kg/d)		2.07 ± 0.02^ab^	2.07 ± 0.02^ab^	2.09 ± 0.02^ab^	2.15 ± 0.03^a^	2.06 ± 0.02^b^	0.039

^1^Predicted means and SEs and P-values from GLMs, where ‘cow age’ and ‘weeks in milk’ were included in all models as categorical factors.

^2^Means that do not share a superscript letter are different at *P* < 0.05.

^3^MY–milk yield; FP–fat percentage; PP–protein percentage; FY–fat yield; PY–protein yield; MSY–milk solid yield.

**Table 3:** Pearson correlation coefficients between milk traits, cow age and weeks in milk (*P* value below).

	**Weeks in milk**	**Cow age**	**MY**	**FP**	**PP**	**FY**	**PY**
MY	−0.307	0.517					
	<0.001	<0.001					
FP	0.046	0.102	−0.374				
	0.218	0.006	<0.001				
PP	0.221	−0.149	−0.511	0.621			
	<0.001	<0.001	<0.001	<0.001			
FY	−0.291	0.598	0.782	0.275	−0.118		
	<0.001	<0.001	<0.001	<0.001	0.002		
PY	−0.256	0.526	0.922	−0.153	−0.146	0.853	
	<0.001	<0.001	<0.001	<0.001	<0.001	<0.001	
MSY	−0.287	0.589	0.873	0.099	−0.135	0.974	0.950
	<0.001	<0.001	<0.001	0.008	<0.001	<0.001	<0.001

MY–milk yield (kg/d); FP–fat percentage (%); PP–protein percentage (%); FY–fat yield (kg/d); PY–protein yield (kg/d); MSY–milk solid yield (kg/d).
